# Enzyme Engineering: Performance Optimization, Novel Sources, and Applications in the Food Industry

**DOI:** 10.3390/foods13233846

**Published:** 2024-11-28

**Authors:** Shucan Mao, Jiawen Jiang, Ke Xiong, Yiqiang Chen, Yuyang Yao, Linchang Liu, Hanbing Liu, Xiang Li

**Affiliations:** 1 Beijing Laboratory for Food Quality and Safety, Beijing Technology and Business University (BTBU), Beijing 100048, China; 2 Beijing Key Laboratory of Flavor Chemistry, Beijing Technology and Business University (BTBU), Beijing 100048, China; 3 Beijing Engineering and Technology Research Center of Food Additives, Beijing Technology and Business University (BTBU), Beijing 100048, China; 4 Beijing Innovation Centre of Food Nutrition and Human, Beijing Technology and Business University (BTBU), Beijing 100048, China

**Keywords:** enzyme preparations, directed evolution, novel sources, immobilization, application

## Abstract

This review summarizes the latest progress in enzyme preparation, including enzyme design and modification technology, exploration of new enzyme sources, and application of enzyme preparation in food processing, detection, and preservation. The directed evolution technology improved the stability and catalytic efficiency of enzymes, while enzyme immobilization technology enhanced reusability and industrial applicability. Extremozymes and biomimetic enzymes exhibit excellent performance under harsh conditions. In food processing, enzyme preparation can improve food quality and flavor. In food detection, enzymes combined with immune detection and biosensors realize rapid detection of allergens, pollutants, and pesticide residues. In food preservation, enzymes enhance food quality by extending shelf life and inhibiting microbial growth. In the future, enzyme engineering will be combined with computer-aided design, artificial intelligence, and new material technology to promote intelligent enzyme design and multifunctional enzyme preparation development and help the technological upgrading and sustainable development of the food industry and green chemistry.

## 1. Introduction

Enzymes are special catalysts in nature, evolved over thousands of generations through natural selection to perform specific biological functions efficiently. A key characteristic of enzymes is the ability to lower the energy barrier of the reaction, selectively promoting and speeding up certain chemical reactions. Enzymatic processes are increasingly valued for their high activity, selectivity, stability, and eco-friendly nature in biocatalysis. In 2019, the global enzyme market size was USD 9.087 billion, and it is expected to grow to USD 13.8152 billion by 2027, with a compound annual growth rate of 6.4% during the period 2020–2027. The report indicates that the demand for bio-based additives in processed food and beverages and growing biotechnology progress promoted the development of the market food enzymes (https://www.fortunebusinessinsights.com/zh/food-enzymes-market-102835 (accessed on 3 October 2024)).

**Figure 1:**
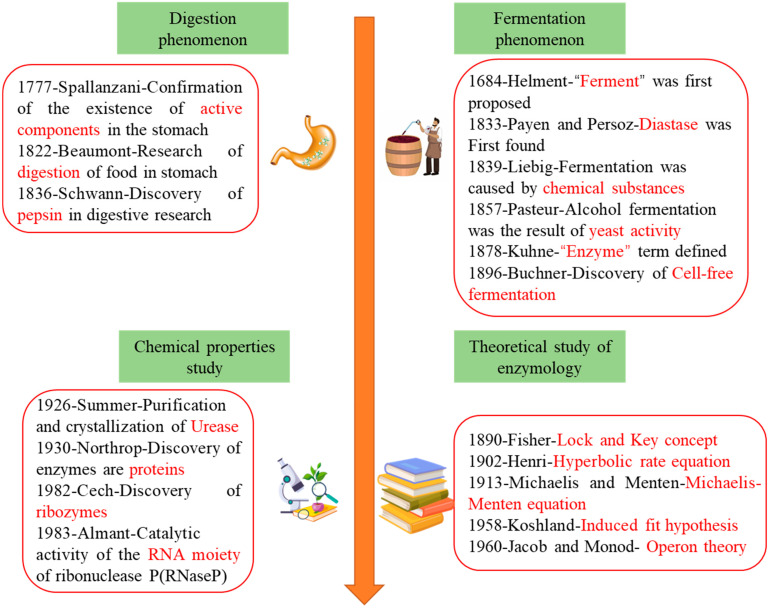
Landmarks in enzyme studies.

Enzymology began in the 19th century with the gradual understanding of the mechanisms of digestion and fermentation. Figure 1 illustrates the development of enzymology research. The use of natural enzymes for food processing and preservation dates back to ancient times, including bread-making in Egypt and fermentation practices across Eastern cultures. Over time, enzyme applications transitioned from traditional practices to systematic scientific research [1,2], revealing their chemical properties [3,4] and establishing the theoretical basis of enzyme engineering [5,6]. Enzyme engineering flourished in the late 20th century with advances in molecular biology and genetic engineering. However, large-scale production faces challenges as harsh solvent systems and mechanical stress often reduce enzymes’ thermal stability and catalytic activity, limiting industrial applications. Furthermore, as researchers deepen their understanding of enzyme structure and function, they increasingly recognize the importance of enzyme performance optimization in enhancing catalytic efficiency and meeting specific industrial demands. Consequently, various optimization techniques have emerged, including genetic engineering [7], chemical modification (such as covalent modification [8], cross-linking [9], and immobilization), directed evolution [10], natural enzyme screening and modification [11], and artificial enzyme development [12]. The exploration of these technologies collectively facilitates the enhancement of enzyme performance, addressing the industry’s need for innovation and quality and laying a solid foundation for the efficient and economical application of enzyme preparations in the food industry. Notably, directed evolution, enzyme immobilization, and new enzyme source exploration offer significant advantages in enzyme performance optimization. Proposed by Frances Arnold in the 1990s [13], directed evolution represents a breakthrough in enzyme engineering. By employing random mutagenesis and screening, this method generates highly adaptable and diverse enzyme variants, even without prior structural knowledge. Building on traditional random mutagenesis and screening, modern directed evolution integrates rational design and computational tools to leverage structural data and functional predictions, optimizing mutation strategies and improving screening efficiency. In contrast, genetic engineering typically requires a deep understanding of the structure and function of the enzyme. Compared with chemical modification methods such as covalent modification and cross-linking, enzyme immobilization significantly enhances the thermal stability and pH stability of enzymes by fixing them onto solid supports, allowing for their repeated use and simplifying the separation process. This enables immobilized enzymes to maintain catalytic activity for extended periods and operate under a wider range of conditions, further promoting their widespread use in large-scale industrial applications. Traditional enzyme sources face challenges like high production costs, complex extraction, and low stability, highlighting the need to explore new enzyme sources [14]. In this process, natural enzyme screening and modification, as well as artificial enzyme development, have become important research directions. These approaches not only expand the sources of enzymes but also enhance their functionality and adaptability. With advancements in these technologies, the application of enzyme preparations in the food industry is poised for a more efficient and economical transformation, driving ongoing innovation and development in the sector. This paper reviews recent advances in directed evolution, enzyme immobilization, novel enzyme sources, and the applications of enzyme preparations in the food industry.

## 2. Performance Optimization Techniques for Enzymes

Enzymes play an essential catalytic role in nearly all biological processes but face limitations, such as restricted stability, substrate range, and productivity, which hinder their industrial applications. To solve these key limitations in biocatalysis, the focus of enzyme research has shifted from enzyme mechanisms to improving enzyme performance or creating new enzymes, paying more attention to the environmental protection and sustainable development of enzymes. Scientists now use enzyme engineering technologies, including rational design, directed evolution, and computer simulation, to design and modify enzyme structures. Enzyme immobilization enhances stability and activity in specific environments, increasing sustainability. This expands enzyme applications and improves stability in complex environments. These innovations overcome natural enzyme limitations and enable the development of new catalysts, showing significant potential in food industry applications. This section will focus on the directed evolution and immobilization techniques for enzyme performance optimization.

### 2.1. Directed Evolution

Directed evolution is a crucial enzyme engineering technique in the food industry, simulating Darwinian natural evolution in the laboratory. Through repeated cycles of mutation and screening or selection, it enables the rapid optimization and enhancement of enzyme performance, significantly shortening the time required compared to natural evolution. Traditional methods like error-prone PCR and DNA shuffling are limited by genome complexity and low mutation rates, resulting in slower evolution and labor-intensive operations, which hinder large-scale experiments [15]. These problems have limited the widespread use of traditional methods in the food industry.

**Figure 2:**
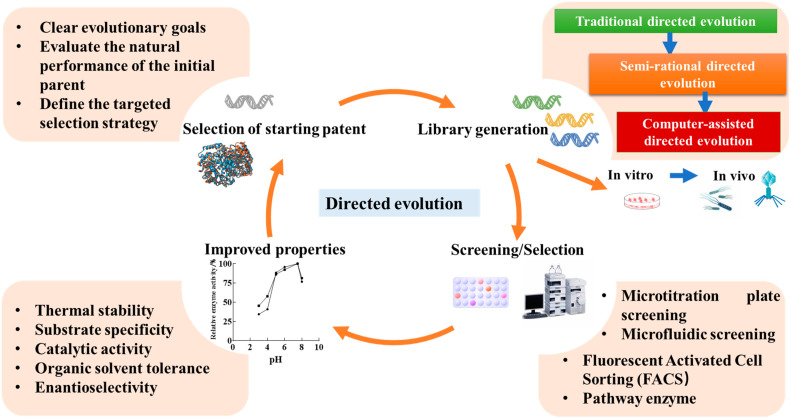
The pathway of directed evolution.

Directed evolution techniques can significantly improve the performance of enzymes used in the food industry, such as substrate specificity [16], enantioselectivity [17], thermal stability [18], and organic solvent resistance [19]. These improvements directly impact productivity, quality, and cost in the food industry. Directed evolution consists of three steps: (1) selecting a starting paternal parent, (2) creating a genetic library, and (3) screening and selection [20]. The preconditions for directed evolution include selecting a highly active enzyme as the starting point, serving as the “patent” in the evolutionary process, defining clear optimization goals, and designing appropriate mutagenesis strategies. Once the library is established, efficient screening identifies enzyme variants with desired properties (Figure 2). This section mainly introduces two steps of genetic library establishment and high-throughput screening, which contribute to the continued development of the food industry.

#### 2.1.1. Genetic Library Creation in Directed Evolution

Each step of directed evolution is critical for enzyme optimization, with genetic libraries determining the diversity of mutants for screening. Thus, designing a high-quality library is fundamental. Different strategies used in library creation can affect the quality and screening efficiency of the library. Several common strategies for creating genetic libraries are summarized in Table 1. Traditional mutagenesis often generates overly large genetic libraries, which lowers quality and complicates screening. With technological advancements, in vivo mutagenesis has gradually shown greater advantages, such as conducting mutation screening in environments closer to real organisms, avoiding enzyme purification, and utilizing high-throughput screening to optimize complex biological pathways. Common hosts for in vivo mutagenesis include bacteria (E. coli [21], Bacillus subtilis [22], Clostridium [23]), yeast [24], mammalian cells [25], and phages [26]. These hosts express different mutated genes and thus produce corresponding mutated enzymes, which lays a foundation for subsequent screening or selection. Several in vivo directed evolution platforms have been developed to optimize specific functions. The OrthoRep system enables rapid in vivo evolution of target genes through a high mutation rate and orthogonal DNA polymerase system without interfering with the host genome [27]. OrthoRep technology can be used in the food industry to enhance the quality characteristics of food products, such as flavor, color, and nutrition, by leveraging plant-derived enzymes [28]. The Mutagenic Organized Recombination Process by Homologous In Vivo Grouping (MORPHING), based on yeast’s natural homologous recombination, facilitates random domain mutagenesis and recombination in target genes, generating diverse mutant libraries [29]. MORPHING has been widely applied in the development and modification of biocatalysts, offering significant advantages in optimizing enzyme performance and expanding their functions. Phage-Assisted Continuous Evolution (PACE) uses phages for rapid, continuous evolution in bacterial hosts, often for complex protein development [30]. Currently, PACE technology is mainly used in antibody production, proteomics, therapeutics, diagnostics (especially for cancer applications), infectious diseases, and drug discovery [31], and with further development, it has potential in other areas such as the food industry. CRISPR/CAS-mediated directed evolution provides a powerful tool for enzyme engineering and gene function optimization, combining site-specific mutagenesis with random mutagenesis to efficiently evolve target genes in vivo. Platforms like EvolvR [32], CRISPR-X [25], and CasPER [33] integrate CRISPR with directed evolution, enabling precise in vivo mutations through CRISPR-guided polymerase systems. These platforms address the different needs for genome editing and mutagenesis. EvolvR, combined with directed evolution (awarded the Nobel Prize in Chemistry in 2020), can rapidly generate libraries of functional gene variants, such as cytochrome P450 or even Cas9. In the context of the human microbiome, microbes are employed as living drugs but often struggle to survive the harsh gut environment. EvolvR-based genome-wide screening offers potential for identifying strains with enhanced intestinal viability [34]. CRISPR-X enables precise local mutagenesis of cytidine deaminase through dCas9 guidance, showing promise in functional gene research and the exploration of drug resistance mechanisms. In contrast, CasPER integrates error-prone PCR with CRISPR/Cas9-mediated genome integration to facilitate the targeted evolution of enzymes within genomic environments. This method has significant potential in the food industry, particularly in enhancing natural product biosynthesis, such as carotenoid production in yeast [35].

Directed mutagenesis increasingly incorporates rational and computer-aided design to improve mutation library quality and screening efficiency. Rational design involves using the literature, modeling, and knowledge of protein structures and sequences to generate novel proteins with desired properties. While the rational methods are powerful and fast, the availability of useful information is a limitation. Rationally designed libraries tend to be small because they are labor-intensive to create, which limits their practicality. Furthermore, proteins are complex and dynamic three-dimensional structures, making it difficult to reliably predict whether these rationally designed modifications will yield the expected results [30]. These limitations often lead to the combination of rational design with random methods like directed evolution to improve the success rate of enzyme optimization. Semi-rational strategies integrate directed evolution with rational design to overcome the limitations of both approaches. Semi-rational design primarily relies on bioinformatics methods, using protein structure, function, sequence homology, and computational prediction to pre-select suitable target sites, followed by focused mutagenesis with limited amino acid diversity. This approach involves the rational selection of multiple amino acid residues for mutation, the use of efficient codons, and the construction of high-quality mutant libraries to specifically engineer proteins. A series of semi-rational libraries have been developed based on structural and sequential information, such as Structure-based Combinatorial Protein Engineering (SCOPE) [36] and combinatorial site-saturation mutagenesis (CSSM) [37]. In addition, strategies like the Combinatorial Active-Site Saturation Test (CAST) and Iterative Saturation Mutagenesis (ISM) are based on site-specific mutagenesis at active sites. These methods aim to generate smaller and more feature-rich mutation libraries. However, they typically require a large amount of accurate protein structural and sequence information. Semi-rational design has proven highly effective in enhancing enzyme performance. Using this strategy, *Bacillus megaterium* M32 carboxypeptidase (CPM32) was modified to improve its catalytic efficiency and thermal stability. The modified enzyme demonstrated improved freshness and reduced bitterness in food flavor modification, highlighting its potential for developing high-quality protein ingredients [38]. With the further development of computer-aided technologies, combining directed evolution with new computational and hybrid methods enables researchers to design “smart” mutation libraries that reduce screening difficulties. For example, the emergence of ProSAR (protein sequence activity relationships), a library optimization tool based on statistical analysis, can significantly reduce the screening workload required for successful directed evolution [39]. ProSAR is a powerful computational framework for linking protein sequence variation to functional traits, enabling rational protein design and optimization in applications such as enzyme engineering and therapeutic development. In addition, KnowVolution is a comprehensive protein engineering strategy that uses computational predictions and experimental validation to prioritize beneficial mutations, creating more targeted and functional gene libraries, which makes the process of screening for beneficial enzyme variants more efficient [40]. KnowVolution enhances enzyme properties, including the specific activity of lipase [41], the alkaline tolerance of fungal laccase [42], and the thermal stability of cellulase [43] by integrating computer-assisted strategies with directed evolution. Moreover, there are already machine-aided design platforms (Rosetta design [44], Transcent [45], IPRO [46], Osprey [47], Alphafold [48]) for enzyme-aided design and functional modification. To better leverage the advantages of computer-aided design technologies, researchers continuously integrate these technologies with traditional directed evolution methods to enhance the directed evolution of enzymes.

Enzyme engineering has evolved significantly, from basic studies of enzyme mechanisms to advanced techniques like directed evolution. Directed evolution accelerates enzyme optimization through iterative cycles of mutation and selection, improving traits such as substrate specificity and stability. Traditional methods like error-prone PCR and DNA shuffling have laid the groundwork, but new in vivo techniques, such as OrthoRep, MORPHING, and PACE, offer enhanced precision and efficiency. Computational tools and semi-rational strategies complement these methods by optimizing mutation libraries and improving screening processes. The future of enzyme engineering will likely see further integration of computational methods with experimental techniques, enabling more rapid and accurate enzyme optimization. Advancements in artificial intelligence and machine learning are expected to drive the development of even more sophisticated predictive tools, enhancing our ability to design and engineer enzymes with highly specific functions. Additionally, the continued exploration of novel enzyme sources and improvements in both in vivo and in vitro mutagenesis methods will expand the scope and efficiency of enzyme engineering across various industries.

**Table 1:** Strategies for creating genetic libraries.

Mutation Type	Mutation Method	Purpose	Advantages	Limitation	Reference
In vitro directed evolution	Error-prone PCR	Random mutation	Easy to perform Does not require prior knowledge of key positions	Reduced sampling of mutagenesis space Mutagenesis bias	[49]
	DNA shuffling	Random sequence recombination	Recombination advantages	High homology between parental sequences required	[50]
	Combinatorial Active-Site Saturation Test (CAST)	Random mutation at active sites based on 3D protein structure	Structure-guided precision High efficiency in screening Reduced screening effort Flexible application	Dependency on structural information Computational requirements High experimental cost Limited by mutation library size	[51]
	Iterative Saturation Mutagenesis (ISM)	Saturation mutagenesis in a specific region of a protein	Focused optimization Accelerated progress Enhanced performance Systematic exploration	Labor-intensive Time-consuming Potential for local optima Dependency on initial selection Computational requirements	[52]
	Site-saturation mutagenesis (SSM)	Specific location-focused mutagenesis	Rapid identification of beneficial mutations Efficient library size	Limited to predefined sites Labor-intensive Requires prior knowledge Saturation limits	[53]
	Combinatorial Coevolutionary Saturation Mutagenesis (CCSM)	Multiple site-specific mutations	Multisite mutation Increased probability of functional improvement	The huge mutant libraries Increased screening difficulty High computational and experimental requirements	[37]
	KnowVolution	Enhance enzyme function by combining rational design and directed evolution	A rational and systematic approach Detailed understanding of molecular mechanisms Efficient mutation identification Iterative improvement	Dependent on available structural data Computational complexity Limited to known mutational effects	[40]
In vivo directed evolution	OrthoRep	Continuous high-mutation-rate evolution of specific genes in vivo	Maintains high mutation rates in targeted genes No disruption to host cell growth or function Continuous and autonomous evolution without manual intervention Ideal for long-term studies Scalable for optimizing complex traits	Maintains high mutation rates in targeted genes Mutation accumulation can be slow for highly complex traits Dependent on the host organism’s ability to express and process the evolved proteins	[27]
	Phage-Assisted Continuous Evolution (PACE)	Continuous evolution method based on phages	High mutation and selection speed Continuous and automated Flexible mutagenesis	Limited to phage-compatible proteins Complex setup Restricted evolutionary environment	[30]
	CRISPR/Cas-mediated directed evolution	Target mutations to accelerate the evolution of enzymes in vivo	Highly specific targeting of genomic loci Real-time evolution in a biologically relevant environment No requirement for pre-existing structural information about the target protein	Potential off-target effects Limited by the efficiency of Cas9 delivery systems Requires optimization of guide RNAs for each experiment	[54]
	Mutagenic Organized Recombination Process by Homologous In Vivo Grouping (MORPHING)	Random domain mutagenesis and recombination of specific protein segments	Precise mutagenesis High fidelity in recombination Suitable for eukaryotic systems	Limited to short protein segments Potential mutational bias due to the use of error-prone PCR, which may require further optimization.	[29]
Other directed evolution	Structure-based Combinatorial Protein Engineering (SCOPE)	Directed mutation based on protein 3D structure integrated with DNA manipulation technology	Structure-guided design High efficiency Directed optimization Broad applicability	Complex workflow High cost Limited by structural information Screening challenge Potential off-target effects	[36]

#### 2.1.2. High-Throughput Screening in Directed Evolution

High-throughput screening and selection are critical in directed evolution for enzyme optimization. Traditional methods include AGAR plate and microtitration plate screening. AGAR plate screening uses visual signals to identify enzyme variants, offering simplicity but low precision [55]. Microtitration plate screening employs UV–Vis absorbance, fluorescence, or colorimetric analysis, providing a broad dynamic range but limited scalability for large libraries [56]. In contrast, selection strategies that rapidly eliminate mutants that do not possess target properties by applying specific pressures (such as antibiotics or nutritional restrictions) have higher throughput but require enzyme properties that are highly correlated with survival [57].

Ultra-high-throughput screening methods have expanded the size of screenable genetic libraries, mainly in the following categories: display technology-based screening, cell-based screening, and computer simulation-based screening. Among these, display technology-based screening is a protein engineering technique often used to improve the binding affinity or stability of proteins. It is generally divided into phage display [58], bacterial display [59], ribosome display [60], mammalian display [61] and yeast display [62]. The direct link between phage DNA and the proteins on display makes it possible to screen for specific phenotypes. Bacterial display involves displaying foreign proteins on the outer or inner membrane of bacteria (such as *E. coli*), achieved by fusing foreign proteins with bacterial membrane proteins. Both bacterial display and phage display lack post-translational modifications, making them unsuitable for displaying proteins that require complex modifications. The above problems are well addressed by yeast display, which is a eukaryotic organism capable of performing complex post-translational modifications. Moreover, the combination of yeast display and Fluorescence-Activated Droplet Sorting (FACS) has high screening efficiency and can be quantitatively screened. Mammalian displays allow for post-translational modifications (such as glycosylation) and display proteins that are more similar to human proteins, thus having important applications in drug discovery and antibody engineering. Compared with bacterial display and bacteriophage display, the screening cost of mammalian display and yeast display are more expensive and complex to operate. Furthermore, the ribosome system can be performed without cells and can screen larger libraries, but it relies on in vitro transcriptional translation systems, making it more susceptible to environmental factors. Each display system has its advantages and disadvantages, and selecting the appropriate method depends on the characteristics of the target enzyme and the experimental needs [63].

Cell-based screening primarily uses fluorescence-activated cell sorting (FACS), magnetic-activated cell sorting (MACS), and Fluorescence-Activated Trickle Sorting (FADS). MACS is used to label cells with antibodies bound to magnetic beads, make the target cells wear magnetic beads, and separate them from non-labeled cells via a magnetic field. However, due to the long operating time, cross-contamination is more likely to occur with this method. To overcome these problems, microfluidic-based magnetic-activated cell sorting (μMACS) was introduced to dramatically reduce reagent use and improve purity, throughput, and continuous flow recovery rates [64]. FACS is a highly efficient single-cell sorting technique based on fluorescent activation, capable of detecting billions of samples [65]. This method is mainly used to detect the content of substances in cells. To solve the problem of high-throughput screening for cell secretory expression, the researchers developed FADS. This method uses droplet microfluidics to generate uniform, independent microdroplets via microchips, encapsulating cells within these droplets for high-throughput detection and sorting of single-cell secreted products. FADS offers high throughput and low cost, making it widely applicable in the discovery and modification of industrial enzymes [66].

Virtual screening is a promising computational design method based on computer simulation. It is an alternative to mutation library experimental screening but can save time and labor. It identifies potential candidates for further experimental validation through rapid searches of large enzyme libraries based on computational simulations. A computation-based metabolic engineering framework called Selection Finder (SelFi) has been developed to identify high-throughput selection to isolate active mutant enzymes with desired catalytic functions. Given the desired enzyme product, SelFi is the first automated method to identify the enzyme activity selection pathway, advancing the directed evolution of enzymes.

**Figure 3:**
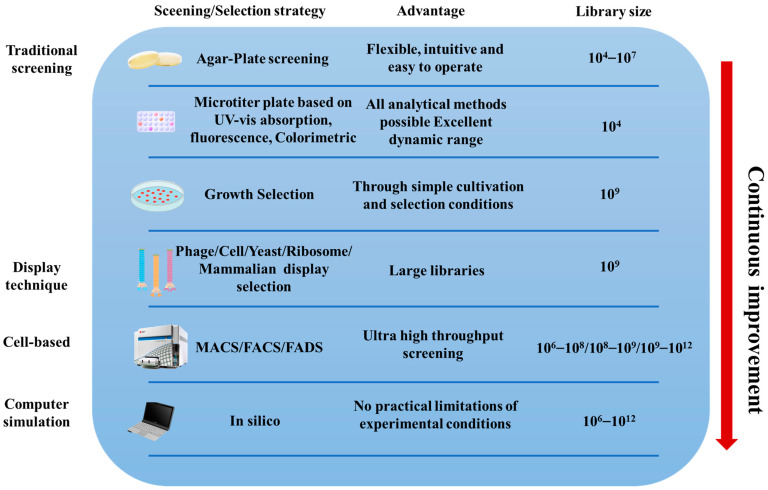
The advantages and the size of genetic libraries that can be screened for high-throughput screening strategies.

In recent years, a variety of high-throughput screening (HTS) methods have been developed to rapidly evaluate genetic libraries, ranging from in vitro screening to in vivo selection, from indicator addition to multi-enzyme system construction, and from plate screening to computational or machine-assisted screening. These techniques have greatly enhanced the efficiency of mutant screening in directed evolution, reduced experimental cycles, and advanced the optimization and modification in the food industry. These screening and selection methods not only greatly facilitate the discovery of novel enzymes, proteins, and small molecules but also provide powerful tools for the field of biotechnology. Moreover, as methods for directed evolution continue to improve, researchers have also developed enzyme immobilization techniques to further optimize enzyme stability, reusability, and catalytic efficiency (Figure 3).

### 2.2. Immobilization of Enzymes

**Figure 4:**
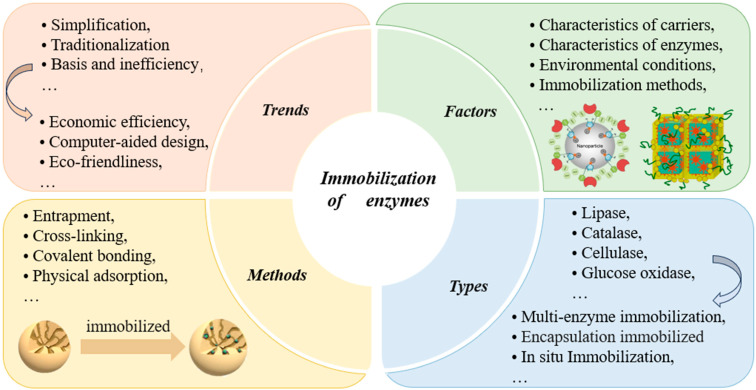
Immobilization of enzymes.

“Enzyme immobilization” refers to the process of localizing or confining free enzymes within a specific region, maintaining their catalytic activity, and allowing for repeated and continuous use [67]. The application scenarios, types, influencing factors, and development trends of immobilization are shown in Figure 4. As previously mentioned, enzyme engineering techniques, including rational design, directed evolution, and computer simulations, play a crucial role in optimizing enzyme performance and complementing enzyme immobilization technology, collectively advancing enzyme optimization and application. Enzyme engineering optimizes the structure and catalytic activity of enzymes by altering their amino acid sequences, and these optimized enzymes can be effectively immobilized onto carriers through immobilization techniques, significantly enhancing enzyme recovery, reusability, and applicability in industrial processes. Ultimately, this leads to more efficient biocatalysis and broader industrial applications. Currently, immobilization technology is advancing rapidly. It supports multifunctional immobilization and can be integrated with computational simulation techniques, allowing for precise control of immobilization conditions and reaction environments. The future is expected to be more intelligent, multifunctional, and sustainable, thus further enhancing the economic viability of enzyme immobilization technology in industry.

In addition, selecting the appropriate immobilization method and carrier is fundamental to achieving efficient and stable biocatalysts. This section introduces various methods and carriers for enzyme immobilization, aiming to provide researchers and industry professionals exploring the application potential of enzyme immobilization technology across diverse areas, such as the food industry and environmental science, with exciting insights.

#### 2.2.1. Traditional Immobilization

Based on the interaction mechanisms between enzymes and carriers, conventional immobilization methods can be categorized into two main types: physical methods (adsorption and entrapment) and chemical methods (cross-linking and covalent bonding). Physical methods refer to the adherence or entrapment of enzymes on the surface or within the matrix of carriers through weak forces such as van der Waals forces and hydrophobic interactions [68]. However, these methods do not establish covalent bonds with the carrier, resulting in relatively weaker binding strength. Chemical methods refer to the immobilization of enzymes by forming covalent bonds with the support material through stronger forces such as ether bonds and thioether bonds. The main method of enzyme immobilization in the chemical industry is the chemical method [69]. For example, covalently immobilizing cysteine proteases derived from natural plants in a continuous packed-bed reactor for treating white wine significantly enhances enzyme efficacy and reduces the haze potential and total protein content of the wine while maintaining the sensory characteristics of the food. This demonstrates the broad potential of immobilization methods in optimizing food processing [70]. However, enzymes are occasionally inactivated or leaked. Therefore, the different methods all have their advantages and disadvantages (as shown in Table 2), and there have been researchers who have combined different immobilization techniques to achieve better immobilization. In practical applications, it is necessary to consider the characteristics of the immobilization method, the nature of the carrier, and other conditions to select the best method to maximize the catalytic potential of the enzyme.

Conventional immobilization carriers can be classified as natural polymers, synthetic polymers, and inorganic. However, not all enzymes are suitable for immobilization on all types of carriers. The functional groups, geometry, pore size, morphology, and loading on the surface of the carrier can significantly affect the activity and stability of the immobilized enzyme [77]. Therefore, the selection of a suitable carrier is essential to achieve optimal immobilization, particularly in processes such as winemaking, fermentation, and food processing. The carrier used for immobilization must meet specific conditions, among which the enzyme-carrier affinity is particularly important. This affinity is achieved by specific reactive groups on the carrier [78], but an overly strong adhesion may adversely affect the performance of the enzyme so that, in some cases, the release of the enzyme from the carrier is necessary. Interestingly, multiple enzymes may be immobilized in the same carrier, and one enzyme can also be immobilized by multiple carrier materials, a phenomenon known as composite carriers and co-immobilized enzymes. They provide more flexible and efficient solutions for catalysis and biotechnology in the food industry. Table 3 summarizes the commonly used carriers for conventional immobilized enzyme technology and their immobilization targets.

#### 2.2.2. New Type of Immobilization

Due to the increasing demands for catalytic efficiency and selectivity in industrial production, coupled with the instability and complexity of traditional methods, conventional immobilization techniques are increasingly inadequate to meet modern production requirements. As a result, innovative immobilization technologies are continuously emerging [93,94,95,96]. These techniques utilize advanced materials or innovative immobilization methods. Furthermore, the latest applications of nanotechnology and materials science are elevating enzyme immobilization technologies to new heights. They not only make the design of carriers more flexible but also enable multifunctional modifications and site-specific immobilization.

Currently, carrier-free biocatalytic systems attract considerable interest due to their advantages, such as not requiring a support or matrix, eliminating the need for matrix modification or activation, and exhibiting minimal leaching effects. Cross-linking, as a traditional enzyme immobilization technique, is characterized by its notable feature of not requiring carrier materials. A common crosslinker is glutaraldehyde, but because it may enter the active site of the enzyme and cause enzyme inactivation, researchers have begun to look for various alternatives, such as chitosan [97]. The two forms of immobilized enzymes obtained through cross-linking are Cross-linked Enzyme Aggregates (CLEAs) and cross-linked enzyme crystals (CLECs). The nucleation mechanism of CLECs and CLEAs is different. CLECs spontaneously nucleate during crystallization, while CLEAs clump enzymes together under solution conditions (pH, salt concentration) and are subsequently cross-linked by the addition of crosslinkers to form cross-linked structures.

Compared to traditional immobilization techniques, CLEAs offer advantages such as straightforward preparation, high stability, and good tolerance to organic solvents, which make their application in the food industry more extensive. For example, in winemaking and fermentation processes, CLEAs can effectively catalyze reactions, optimizing flavor and texture. Their good organic solvent tolerance allows for flexible application in various food environments. Furthermore, the simplicity of the preparation process reduces the production costs of CLEAs, further promoting their adoption and application in the food industry. Additionally, this technology can immobilize different enzymes to form “combinatorial cross-linked enzyme aggregates (combi-CLEAs)”, thereby enhancing reaction efficiency and selectivity. However, CLEA faces challenges such as low mechanical strength, mass transfer limitations, and the need for precipitants and cross-linking agents. Notably, most of these challenges can be addressed through process optimization [98], improvements [99], and protein engineering techniques [100]. Mohd et al. enhanced cross-linking with glutaraldehyde by introducing additional lysine residues to xylanase [100]. The results indicated that the mutated xylanase CLEA exhibits higher stability and reusability compared to the original CLEA. To date, CLEA has been successfully applied to the immobilization of various enzymes, including but not limited to different hydrolases and lyases, which play an important role in the food industry [76]. Cross-linked enzyme crystals represent a novel type of immobilized biocatalyst developed in the 1980s and 1990s. In 1964, Quniocho et al. successfully prepared cross-linked enzyme crystals of carboxypeptidase [101]. Compared to soluble enzymes, cross-linked enzyme crystals demonstrate lower mass transfer limitations and better resistance to denaturation caused by extreme conditions.

Covalent immobilization, as a traditional immobilization method, involves the reaction between enzyme side chain groups and surface groups of a carrier, forming covalent bonds. However, the inability to control the orientation of enzyme attachment on the surface may lead to a loss of enzyme activity [102]. In contrast, the improved site-specific covalent immobilization techniques utilize pre-designed binding sites, selecting specific amino acid residues for immobilization. This approach allows for precise enzyme immobilization at targeted locations, maximizing its structural integrity and functionality. This technique primarily relies on non-covalent strategies. Jeffrey et al. first demonstrated site-specific covalent immobilization of enzymes at residues throughout the enzyme in 2015 [71]. Currently, such methods have been applied to virus detection [103] and biotransformation [104], among other areas. Moreover, co-immobilization can be combined with directional immobilization to form directional co-immobilization.

However, while site-specific covalent immobilization effectively preserves enzyme structure and function, it may provide only single binding points in some cases, limiting the enzyme’s spatial conformation and catalytic efficiency. In contrast, Multipoint Covalent Attachment (MCA) can immobilize enzymes at multiple specific locations, thereby enhancing stability and catalytic efficiency. This method shows promise for improving the operational performance of enzymes as industrial catalysts [101]. Unfortunately, this approach is suitable only when the enzyme surface contains lysine residues [105]. To overcome this limitation, chemical or genetic amination of protein surfaces has proven to be a useful strategy. In a recent study, Godoy et al. immobilized a modified BTL2 mutant with exposed cysteine residues on glyoxyl-disulfide (Gx-DS) using site-specific MCA combined with genetic and/or chemical amination [106]. This notably increased the enzyme’s stability, productivity, activity, and selectivity, further supporting MCA as an effective method for optimizing enzyme catalysts while also broadening the application prospects of this enzyme in the food industry, particularly in areas such as flavor enhancement.

**Figure 5:**
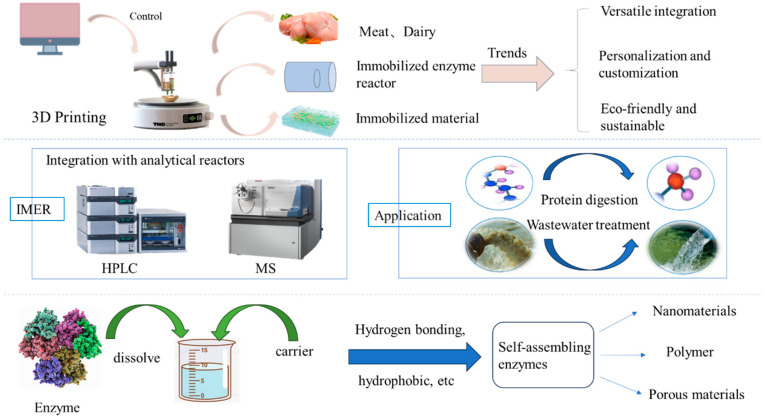
A new type of immobilization technology that is independent of conventional immobilization technology.

Based on the success of the new immobilization technology, researchers have continued to explore and develop related technologies (as shown in Figure 5). These technologies not only retain the advantages of traditional immobilization methods but also innovate on these technologies, making them more competitive and adaptable in modern applications. Three-dimensional printing technology, as an emerging technique that integrates multiple disciplines such as engineering and computer science, enables the precise design and manufacture of porous carrier materials for immobilization, such as Metal–Organic Frameworks (MOFs), polylactic acid (PLA), and hydrogels [107]. This opens new avenues for enzyme immobilization technologies. Depending on the underlying printing principles, methods can be categorized into Stereolithography (SLA), Fused Deposition Modeling (FDM), and extrusion-based 3D printing [75]. Compared to traditional molding and subtractive manufacturing, 3D printing offers greater flexibility and accuracy in rapidly producing complex structures [93]. However, not all carrier materials are compatible with 3D printing; generally, materials must exhibit good rheological properties. Shao et al. proposed a novel continuous flow bioreactor that is immobilized on a 3D-printed scaffold containing β-galactosidase (β-Gal), successfully producing lactose-free milk and achieving certain economic and social benefits [108]. This demonstrates the innovative application potential of 3D printing in food processing. Furthermore, with technological advancements, 4D printing products are emerging, capable of responding to external or internal stimuli over time, thereby altering their physical properties [109]. Exploring food-grade materials for 4D printing has become a prominent research direction within the food industry.

The Immobilized Enzyme Reactor (IMER) represents a system that retains enzymes on designated carriers within the reactor, effectively catalyzing reactions. This system can also be defined as a reusable flow apparatus. Based on structural and operational differences, IMERs can be categorized into microfluidic reactors [110], membrane reactors [111], fixed-bed reactors [112], and fluidized-bed reactors [113]. Immobilizing enzymes within reactors not only minimizes diffusion distances but also enhances mass transfer efficiency, boasting high flux and reduced analytical times [114]. Moreover, the flexible applicability of Immobilized Enzyme Reactors enables them to meet various food processing needs, making them essential tools for achieving efficient and sustainable food production. Currently, IMER technology finds applications in various fields, including proteomics [115], inhibitor studies [116], and industrial production [111]. However, its most common use remains in conjunction with analytical instruments such as high-performance liquid chromatography to facilitate the detection of fragments or product molecules. Despite its potential, the broader adoption of IMERs within analytical and industrial domains faces difficulties, including reproducibility in IMER production, robust integration into analytical platforms, and residual sample issues [114]. In the future, this technology is expected to evolve into more intelligent, multifunctional, and environmentally friendly systems, integrating nanomaterials to significantly enhance reaction efficiency and selectivity, thereby promoting sustainable development.

Assembly immobilized enzyme is a method to immobilize an enzyme by dissolving the enzyme molecule and mixing it with a self-assembled precursor, using its non-covalent interactions, such as electrostatic and hydrophobic interactions, to make the enzyme spontaneously form an ordered immobilized structure. This method is often combined with other technologies to enhance its performance and expand its range of applications. It also offers good controllability, wide applicability, and high enzyme utilization efficiency. Zhuang et al. improved the production efficiency of gluconic acid through self-assembly co-immobilization of glucose oxidase (GOD) and catalase (CAT), demonstrating the potential for the catalyst to maintain high activity during repeated use, thus providing a more economical and environmentally friendly production solution for the food industry [117]. Moreover, self-assembly is a widespread natural phenomenon that involves a variety of substances and systems from molecular to macrostructural levels, mainly including molecular self-assembly, nanomaterial self-assembly, and macrostructural self-assembly [118,119,120]. Inspired by biological systems, self-assembly is usually regulated by the formation or disassembly of self-assembly building blocks catalyzed by coupled enzymes. Many enzymes have been studied for self-assembly, highlighting the potential of utilizing localized biocatalytic self-assembly to achieve complex (biological) functionality, providing an effective approach for the development of smart biomaterials [96].

**Figure 6:**
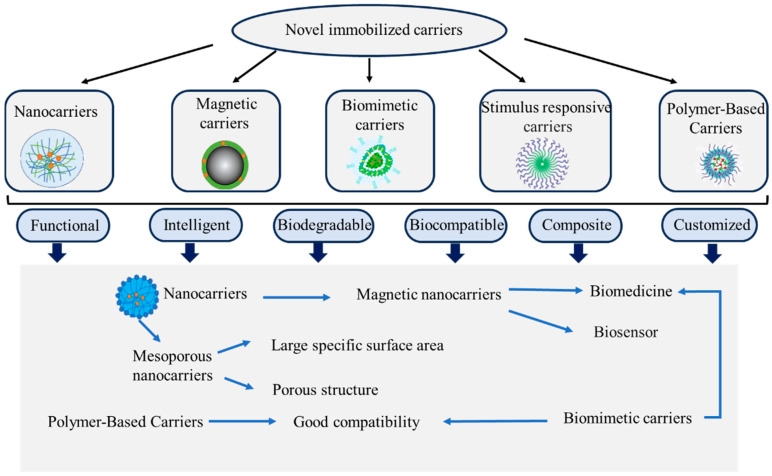
Novel immobilized carriers.

The application of novel immobilization methods has significantly enhanced the immobilization efficiency and catalytic activity of enzymes, providing a solid theoretical foundation and empirical support for the development of new immobilization carriers. In addition, advancements in bioengineering, along with the demands from emerging fields, have led to the emergence of novel carriers like nanomaterials, polymers, and microspheres. These developments provide more options for enzyme immobilization (as shown in Figure 6). In the future, computational simulations and machine learning should be utilized to optimize the immobilization process, enhance immobilization efficiency and enzyme performance, and accelerate the development and application of novel immobilization technologies, thus advancing scientific research and industrial translation.

## 3. Novel Enzyme Source

Modern enzyme engineering techniques, such as directed evolution and enzyme immobilization, have significantly improved enzyme performance, including catalytic activity, stability, reusability, and operational efficiency. However, the application of these optimization techniques relies on abundant enzyme resources as a foundation. To address the increasing demand for efficient enzymes, discovering new enzyme sources is essential. Nature, especially the oceans, an under-exploited ecosystem, harbors many enzymes with unique catalytic properties. In addition, the development of biomimetic enzymes has provided new ideas to address the limitations of natural enzymes, and they exhibit catalytic properties that are even superior to those of natural enzymes under certain conditions. The exploration of enzyme sources and the development of biomimetic enzymes not only provide a breakthrough for modern enzyme engineering technology but also lay the foundation for the innovative development of the food industry.

### 3.1. Novel Enzymes Found in Nature

Enzymes, as biological catalysts, promote almost all life activities in nature. With advancements in technology, humans have explored nature more deeply, especially since many extremophiles have been found in extreme environments. Extremozymes, derived from extremophiles, are highly valued in the industry for their exceptional resistance to extreme conditions. Through active bioprospecting for extreme environments and/or genetic engineering, it is now possible to discover and develop extremozymes that can be adapted to existing industrial processes or products. Compared to traditional enzymes, extremozymes can speed up chemical reactions and reduce the energy requirements of industrial processes, thus saving costs and increasing efficiency. In addition, these enzymes possess novel properties, including tolerance to organic solvents, chiral selectivity, or the ability to degrade complex and stubborn substrates.

Marine ecosystems, covering 71% of the Earth’s surface with an average depth of 3800 m, host the planet’s largest microbial population [121]. The marine environment is extremely complex, featuring extreme conditions such as low and high temperatures, high hydrostatic pressure, strong acidity, and strong alkalinity, accommodating diverse microbial communities adapted to this challenging environment. Table 4 summarizes the types, living conditions, adaptation mechanisms, and major bacterial genera of marine extremophiles. Deep-sea hydrothermal vents host hyperthermophiles (80–150 °C), which are key sources of thermostable enzymes used in processes like starch processing, cellulose degradation, and proteolysis [122,123,124,125,126]. In addition, a new type of thermostable nitrilase was isolated from the hyperthermophilic archaeon *Pyrococcus* sp., showing high stability in terms of temperature, substrate, and product concentration. Artz et al. obtained a heat-resistant mercury ion reduction enzyme from a new hyperthermophile bacterium *Metallosphaera sedula*, which remains active after prolonged incubation at temperatures above 90 °C, with a unique mechanism for coordinating mercury ions [127]. In recent years, research on hyperthermophilic microorganisms has advanced, with researchers constructing a metagenomic library from deep-sea hydrothermal vent samples, leading to the discovery of novel thermostable enzymes. Through directed evolution, these enzymes have been optimized for broader industrial applications, maintaining activity in high-temperature and high-salinity environments [128].

Psychrophiles, which thrive in deep-sea cold environments, exist in all three domains of life (archaea, bacteria, and eukaryotes). Psychrophiles can grow and maintain active metabolism at low temperatures from −20 °C to +20 °C. Cold-active enzymes from psychrophiles are exceptional biocatalysts, offering high specific activity and conformational flexibility at low temperatures but with reduced thermal stability. Many cold-active enzymes, such as lipases and proteases, have been identified, along with pectinases, amylases, and cellulases, all of which have numerous biotechnological applications in food processing [129]. In addition, organisms living in extremely cold conditions can also be potential sources of cold-active enzymes, such as carbonic anhydrase from bony fish [130] and chymotrypsin from Atlantic cod [131]. Furthermore, by using mesophilic or thermophilic enzymes as a parent and engineering them for higher activity at lower temperatures, it is possible to create cold-active enzymes while retaining the stability of the parent [132]. Deep-sea environments also feature high hydrostatic pressure (>110 MPa), and Barophiles are organisms that survive under these high-pressure conditions without breaking down. Many Barophiles grow in the dark for long periods and are often also psychrophiles. The potential biotechnology applications of Barophiles have received little attention compared to other extremophiles [133].

In certain regions of the ocean, microorganisms are usually highly acidophilic or alkaliphilic, capable of surviving at pH < 1 or pH > 11. The extracellular enzymes secreted by these microorganisms are usually acidophilic (optimal pH < 3.0) or alkaliphilic enzymes (optimal pH > 9.0). Compared to neutral enzymes, enzymes functioning at extreme pH levels exhibit good stability in their environments due to a high proportion of acidic or basic amino acids in their molecular structure. Recent studies have also identified acidophilic microorganisms in human-made environments with emerging acidic conditions, such as in corroded concrete sewers affected by environmental hydrogen sulfide (H_2_S), where microbial communities with pH values of 2–4 were observed [134]. Acidophiles often exhibit additional traits like thermophilia, halophilia, or heavy metal tolerance. Enzymes stable under acidic conditions have various applications in the starch, baking, juice processing, animal feed, and pharmaceutical industries, some of which have been commercialized [135]. Alkaliphilic enzymes are rich in arginine and histidine residues, low in glutamate residues, and tend to have a higher isoelectric point. Alkaline cellulase (produced by *Bacillus* sp.) was the first basophilic enzyme reported. In addition, alkaline proteases, cellulases, and lipases are commonly used in detergents [136].

In the ocean, the average salinity of the water is about 3.5%, but it can be higher in some areas (salt lakes, salt pans, salt marshes, etc.). A large number of salt-tolerant or halophilic microorganisms (archaea and eukaryotes) remain stable at high salt concentrations, and halophilic archaea can even survive salt concentrations near saturation levels. Some proteases of halophilic archaea are also alkaline proteases, which function at higher pH values. Additionally, some of these proteases remain stable at high temperatures or in organic solvents and can withstand a variety of surfactants, reducing agents, and both polar and non-polar solvents [137]. Furthermore, halophiles are proving to be promising cell factories for the Next Generation of Industrial Biotechnology (NGIB), and researchers are using genetic manipulation systems to advance the biotechnological potential of halophiles and promote their sustainable and more efficient use [138].

Deep-sea microorganisms represent a promising resource for the modern enzyme industry. Extremophiles and their enzymes, valued for their stability and activity in extreme conditions, have gained significant attention for industrial applications. The ocean, as the largest unexplored ecosystem, harbors abundant extremophilic microbial resources. For example, hyperthermophiles from deep-sea hydrothermal vents can produce enzymes for cellulose and fat degradation in extremely high-temperature environments. On the other hand, psychrophiles from deep-sea cold environments can produce cold-active enzymes, which are widely used in food processing. Additionally, acidophilic, alkaliphilic, and halophilic microorganisms in the ocean secrete stable enzymes under extreme pH and high-salinity conditions, demonstrating their potential for various industrial processes. While extremozymes offer numerous advantages, challenges remain in scaling their production from laboratory research to commercial applications, including optimizing culture conditions and genetic engineering techniques.

**Table 4:** Types, living conditions, and main bacteria genera of marine extremophiles [139].

Microorganism Species	Growth Conditions	Adaptations	Bacterial Genus
Hyperthermophiles	80~150 °C	The structure of fatty acids was used to change the composition of cell membranes.	*Pyrococcus furiosus*, *Thermococcus kodakarensis*, *Methanopyrus kandleri*, *Thermus aquaticus*, *Pyrobaculum aerophilum*
Psychrophiles	−20 °C to −20 °C	The composition of the plasma membrane was changed by the increase in the amount of unsaturated fatty acids.	*Pseudoalteromonas* spp., *Psychrobacter* spp., *Colwellia psychrerythraea*, *Shewanella* spp., *Flavobacterium* spp., *Marinomonas* spp., *Alteromonas*, *Algoriphagus.*
Halophiles	1%~30%	Halophiles regulate adaptation by accumulating and synthesizing compatible solutes that act as intracellular stabilizers or stress buffers or by flowing potassium ions (K+) into the cytoplasm to balance osmotic pressure.	*Dunaliella salina*, *Klebsiella pneumoniae*, *Vibrio carchariae*, *Bacillus circulans STB01*, *Haloferax lucentensis GUBF2*, *Haloferax mediterranei*, *Halobacterium sp. NRC-1*
Acidophiles	pH −0.06~1.0	Adaptations in acidophiles include reduced membrane pore size, proton efflux mechanisms (antiporters, symporters, and H + ATPases), and the accumulation of buffering amino acids.	*Sulfolobus solfataricus*, *Thermoplasma*, *Thermogymnomonas*
Alkaliphiles	pH > 11	The electrogenic, secondary cation/proton antiporters were used to maintain cytoplasmic pH homeostasis and uptake of H.	*Arthrobacter*, *Natronobacterium*, *Psychrobacter*, *Vibrio.*
Barophiles	>100 MPa	Barophiles adapt to high-pressure environments by adjusting lipid composition of cell membrane, stabilizing protein structure, and regulating metabolic pathways.	*Halomonas salaria*, *xenophyophores*

### 3.2. Artificial Biomimetic Enzyme

Building on the discovery of extremozymes from deep-sea environments, another promising avenue in enzyme technology is the development of biomimetic enzymes, also known as artificial enzymes. These are molecules or materials designed and synthesized by mimicking the structure and functions of natural enzymes [140]. These biomimetic enzymes can exhibit catalytic activities analogous to those of natural enzymes under certain conditions, even surpassing the performance of their natural counterparts. Their production processes are relatively straightforward and endowed with remarkable biocompatibility. Beyond these attributes, certain biomimetic enzymes also demonstrate high enzyme-like activities, thereby overcoming the limitations of traditional enzyme mimics, such as high manufacturing and maintenance costs, as well as performance instability [141] (As shown in Table 5). Currently, biomimetic enzymes have found applications in diverse fields, including biomedicine, environmental remediation, industrial catalysis, artificial photosynthesis, and the food industry.

Biomimetic enzymes can be divided into two main categories: metal complex biomimetic and nanomaterial biomimetic. Metal complex biomimetic enzymes use the complex synthesized by metal ions and organic ligands as the catalytic center to mimic the active site of natural enzymes. Such biomimetic enzymes usually have REDOX catalytic functions. The common metal complexes are metalloporphyrins, which are frequently used metal complexes and are the active centers of oxidoreductases. It is formed by replacing two pyrrole protons (N-H) in the porphyrin molecule with metal ions. Bin et al. used the REDOX active center of metal porphyrin, combined with materials (carbon nanotubes), to significantly improve the sensitivity and stability of electrochemical sensors [142]. So far, several methods have been developed to modify the structure of metalloporphyrins to enhance their catalytic activity or stability. Among them, Metal–Organic Frameworks (MOFs), a novel type of fluorescent group assembled from organic ligands and metal ions, endow MOFs with excellent optical properties and biomimetic protease activity [143]. Xu et al. significantly enhanced the catalytic performance and stability of metalloporphyrins as metal complexes by precisely designing atomic-level active sites and regulating the coordination environment within MOF materials [144].

Nanozymes are nanoscale materials that exhibit intrinsic enzymatic activity, characterized by diverse morphologies, high porosity, and chemical stability. Through specific chemical modifications, they can be tailored to demonstrate hydrolytic activity [145]. Reported nanozymes include mimics of peroxidase, superoxide dismutase, laccase, oxidase, and phosphatase. Research on nanozymes has further evolved from the foundational work on metal complex biomimetic enzymes, employing nanoparticles composed of metals (such as Au, Ag, Pt, and Pd) [146,147,148,149], metal oxides (including cerium oxide, zirconium dioxide, and iron oxide) [150,151,152], and transition metal phosphates [153,154,155]. These nanomaterials uniquely combine the properties of metal complexes with those of nanomaterials, offering enhanced catalytic functions. Among nanozymes, those based on metal nanoparticles are particularly notable due to their large surface area and strong catalytic activity, which have led to broad applications. In recent years, gold nanoparticles (Au NPs) have been extensively studied as enzyme mimics. Various surface-modified Au nanoparticles have been synthesized, and they have demonstrated either glucose oxidase (GOx)-like activity or peroxidase-like activity [156]. For instance, citrate-capped gold nanoparticles exhibit GOx-like activity, catalyzing the oxidation of glucose. Meanwhile, cysteine-capped gold nanoparticles, Au nanoparticle/graphene hybrids, and bimetallic bismuth–gold nanoparticles have shown high catalytic activity for the oxidation of H_2_O_2_ [157].

Metal oxide nanomaterials are widely recognized for their excellent environmental compatibility, making them ideal for detecting and degrading environmental pollutants. Cerium oxide (CeO_2_) is considered a green enzyme mimic due to its ability to mimic phosphatase and urease activities, enabling the rapid degradation of organophosphorus toxins [158] and the catalysis of urea decomposition [159]. Zirconium dioxide nanoparticles (ZrO_2_) exhibit exceptional phosphatase-like activity [152], while iron oxide nanoparticles (Fe_3_O_4_) significantly enhance the hydrolysis of phosphate esters. Transition metal phosphates are particularly favored by researchers due to their low cost and ease of availability, often serving as effective substitutes for superoxide dismutase (SOD). This is because transition metal phosphates share a similar catalytic center structure with natural SOD, demonstrating high enzymatic catalytic activity, and the phosphate structure provides stability to redox pairs. Nickel phosphate nanomaterials exhibit high electrocatalytic activity, enabling sensitive detection of superoxide anions from living cells [154]. Additionally, cobalt phosphate and manganese phosphate are also considered good substitutes for superoxide dismutase [153,155].

Two-dimensional nanomaterials, such as graphene, graphene oxide, and black phosphorus, offer advantages like excellent conductivity, stability, and low-cost synthesis, making them suitable for diverse applications. Currently, these materials are primarily used for the detection of toxic metal ions in food and environmental samples [160].

**Table 5:** Classification, advantages, and applications of bionic enzymes.

Type	Enzyme mimics	Materials	Advantages	Methods	Application	Reference
Nanomaterial biomimetic enzymes	Glucose oxidase	Au NPs	High stability, easy preparation and purification, low cost, wide application range	Sodium citrate reduction method	The detection of glucose in serum samples	[146]
	Catalase; peroxidase; superoxide dismutase	Pt NPs	Pt nanoparticles exhibit different biomimetic enzyme functions at different pH conditions	/	It is used in fuel cells, air purification, and anti-aging cosmetics	[147]
	Oxidase; peroxidase	Pd NPs	High catalytic activity, good stability, and selectivity	Hydrothermal method	Detection of heavy metal ions (Pb²^+^)	[148]
	Peroxidase	Ag NPs	Excellent catalytic performance, easy synthesis, and high stability	Hydrothermal method	The chiral recognition of tryptophan enantiomers	[149]
	Laccase	Copper-based dual-ligand nanozyme	Excellent stability under harsh conditions and excellent recyclability. Compared with other laccase mimetic enzymes, Cu-BH exhibits unique fluorescence properties and good biocompatibility	Hydrothermal method	The detection of kanamycin antibiotics	[161]
	SOD (superoxide dismutase)	Nickel phosphate nanoparticles	High enzymatic catalytic activity, low cost, easy fabrication, and nickel phosphate structure provide stability for REDOX	Hydrothermal method	Highly sensitive detection of superoxide anions released from living cells	[154]
	SOD (superoxide dismutase)	Cobalt phosphate nanoparticles	Biocompatibility, unique catalytic and electronic properties	Micro-emulsion method	Highly sensitive detection of superoxide anions released from living cells	[153]
	SOD (superoxide dismutase)	Manganese phosphate nanoparticles	High-efficiency catalytic performance, good biocompatibility	The co-precipitation method combined with freeze-drying technology	Highly sensitive detection of superoxide anions released from living cells	[155]
	Oxidase	Iron nanoparticles	High oxidase-like activity	Self-assembly and pyrolysis processes	The electrochemical detection of dopamine and uric acid in human serum	[162]
Metal complex biomimetic enzymes	Oxidase	Metalloporphyrin	The problem of poor stability of native enzymes was solved	Hydrothermal method	The detection of phenolic antioxidants in vegetable oils	[142]
	Oxidase	Mof-based biomimetic metal porphyrins	Efficient catalysis, enhanced stability, and functional diversity	Solvothermal method	Photocatalysis and artificial photosynthesis	[144]

## 4. Application of Enzyme Preparations

**Figure 7:**
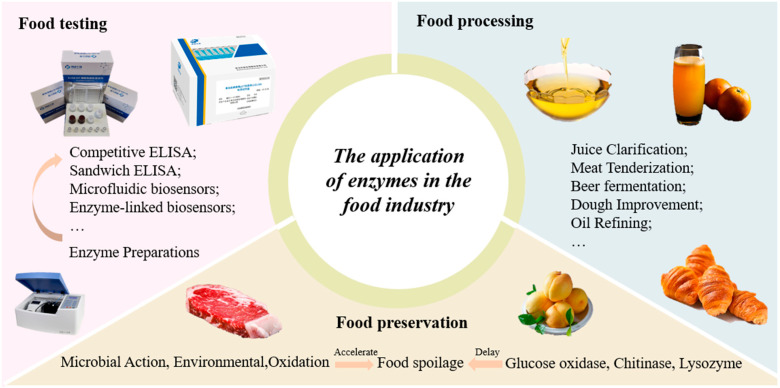
The application of enzymes in the food industry.

Enzyme preparations consist of active enzymes. Due to their specificity and efficiency, they find extensive applications in various fields, including the food industry, agriculture, and biotechnology. In food preservation, enzymes inhibit microbial growth and delay spoilage, thus extending shelf life. In food processing, enzyme preparations enhance production rates, improve texture, and elevate flavor profiles. Furthermore, enzymes play a critical role in food testing, enabling rapid detection of specific components or contaminants through enzymatic reactions, thereby ensuring food safety. Consequently, enzyme preparations hold an indispensable position in the modern food industry. Figure 7 shows an overview of the application of enzyme preparations in the food industry.

### 4.1. Food Testing

Globalization and complex food supply chains have increased the risk of contaminants such as pesticide residues, antibiotics, foodborne pathogens, and toxins, posing a major threat to food safety. The food matrix is extremely intricate, making the diversity of detection methods crucial. Traditional detection techniques, while effective, are time-consuming and demand strict conditions. Modern detection technologies, such as high-performance liquid chromatography, Mass Spectrometry, and Gas Chromatography, not only achieve high sensitivity but also exhibit rapid and on-site testing capabilities, enabling quick identification of potential risks to safeguard consumer health. However, among many detection methods, enzyme-based assays stand out for their high specificity and sensitivity, allowing for the rapid and accurate detection of harmful components in food. Enzyme immobilization further enhances the performance of these enzymes, advancing their application in the food industry. Therefore, this section reviews commonly used enzyme-based detection technologies, aiming to promote ongoing innovation in food safety and quality control methodologies.

#### 4.1.1. Enzyme-Linked Immunoassay

Enzyme-linked immunoassay (ELISA) is an immunological method that uses the affinity of antigens and antibodies and enzyme catalysis to convert substrates into measurable signals (such as color change) for quantitative or qualitative analysis of allergens. This method is characterized by high sensitivity and ease of operation, with the generation of specific signals relying on the catalytic activity of immobilized enzymes such as horseradish peroxidase (HRP) or alkaline phosphatase (ALP). However, these enzymes have some limitations, including instability, complexity of preparation, and high cost. In the face of these cases, nanomaterials also offer a potential way to improve the performance of ELISAs [163]. ELISA methods include competitive and sandwich ELISA, depending on the target analytes and antibodies used [164]. To improve sensitivity, advancements like nanomagnetic bead-based ELISA (NMB-ELISA) and aptamer-integrated ELISA have been developed. These enhanced methods exhibit improved precision and efficiency in detecting target molecules within complex samples, holding significant promise for applications in food safety monitoring. Hu et al. have integrated PCR with the ELISA method [165]. The combined approach is effective for detecting Salmonella and E. coli O157:H7 in diverse food matrices and demonstrates high-throughput testing capabilities. This hybrid technique holds broad potential applications in clinical diagnostics of foodborne diseases, food safety monitoring, and pathogen detection in food.

Currently, the ELISA method’s application in food safety primarily hinges on commercial kits and has been utilized for the detection of allergens in foods such as walnuts and milk, as well as pesticide residues in crops [166,167,168]. However, there are certain limitations in the use of ELISA for practical food testing, including the impact of sample complexity on results, reduced sensitivity, and the potential occurrence of false positives [169,170,171]. Additionally, ELISA demonstrates limited capability for detecting thermally unstable compounds, which may hinder its ability to accurately quantify target components in certain high-concentration samples. As a result, methodologies utilizing specific DNA fragments as detection targets, such as qPCR, biosensors, and high-performance liquid chromatography, have been proposed as alternatives to ELISA. Compared to traditional PCR methods, qPCR monitors fluorescent signals in real time during the PCR process, allowing for dynamic observation of amplification and demonstrating significant advantages in sensitivity and specificity. This technology is primarily applied to the detection of allergens in specific nut foods such as peanuts, sesame, and pistachios, as well as crustacean allergens [172,173,174].

#### 4.1.2. Biosensor Method

Biosensors are analytical systems composed of biomolecular recognition elements and signal processing components, allowing for the specific detection of target substances through processes such as biomolecular recognition, signal transduction, signal amplification, signal detection, and data processing. Depending on their biomolecular recognition elements, biosensors can be categorized into various types, including enzymes, antibodies, nucleic acids, whole cells, and molecularly imprinted polymers. Enzymes, as specific recognition elements, exhibit advantages like high specificity, high sensitivity, rapid response, and environmental friendliness.

Enzymatic biosensors leverage immobilized enzymes to detect target substances in real time via electrochemical or optical methods, enhancing detection speed. Their high sensitivity and rapid response characteristics have increasingly positioned them as essential tools in the field of food analysis. In addition, sensitivity can be enhanced by the addition of substances such as gold nanoparticles (AuNPs) [175]. The initial application of enzymatic biosensor methods involved the quantitative determination of glucose levels in serum; however, they have now been extended to various domains, including food safety assessments, medical diagnostics, and environmental monitoring [164,176,177]. Shavronskaya et al. initially employed β-galactosidase to hydrolyze lactose, releasing glucose and galactose [178]. Subsequently, glucose oxidase was utilized to oxidize glucose, producing hydrogen peroxide (H_2_O_2_), which reacted with a TiO_2_ matrix to generate a yellow peroxotitanium complex detectable via optical methods. The results demonstrated that this system is accurate, sensitive, relatively inexpensive, and easy to implement, requiring minimal sample preparation.

With the development of biotechnology and materials science, the combination of enzyme sensors with nanomaterials, polymers, and biomaterials has given rise to a variety of new sensors. Examples include nanozyme-based microfluidic biosensors [179], novel SRCA-NEMA-G-quadruplex-based electrochemical biosensors [180], and paper-based enzyme-linked biosensors [181]. These innovations not only improve its sensitivity and robustness but also broaden the field of applications, such as allergen detection, pesticide residue testing, and histamine detection [182]. Future advancements in enzyme biosensors will focus on multiplex detection, portability, smart technology, and integration with artificial intelligence and big data, ensuring safer environments and healthier lifestyles. Bai et al. established a portable sensor based on magnetic separation and enzyme-catalyzed nanomaterials that could detect Listeria monocytogenes in food samples as low as 10^−1^ CFU/mL, thereby enabling the immediate detection of Listeria monocytogenes in food samples [183].

### 4.2. Food Processing

Food processing involves transforming raw materials into edible products, encompassing raw material selection, processing, packaging, and storage transportation. In this process, enzyme preparations play a crucial role. As biocatalysts, specialized enzyme preparations typically have a single function to accelerate a specific reaction, while compound enzyme preparations are a combination of enzymes that catalyze multiple reaction processes at the same time, enabling more complex processing needs. By precisely regulating enzymes, food processors can optimize production processes, reduce the use of chemical additives, and reduce costs.

#### 4.2.1. Improve Quality

Food texture is a critical sensory attribute that determines quality and significantly influences consumer acceptance [184]. Optimal textural attributes enhance processing efficiency and improve formability and production efficacy. In this process, enzyme preparations play an irreplaceable role by breaking down specific components or facilitating chemical reactions, thereby improving the texture of food.

Specialized enzymes are highly effective in specific food processing applications. Turbidity in beverages affects taste and quality, leading to significant industrial losses. The mechanisms of turbidity formation mainly include pectin forming micelles, starch swelling to produce gel-like substances, tannins binding with proteins to form insoluble complexes, and protein denaturation resulting in sedimentation. Compared to other chemical treatment methods, enzyme preparations selectively hydrolyze these substances, reducing beverage turbidity and enhancing clarity and quality. Common enzyme preparations include pectinase, amylase, xylanase, tannase, cellulase, and maltase. For example, recently extracted heat-stable endo-pectinase from *Aspergillus niger* has been shown to maintain high stability and activity under high-temperature and alkaline pH conditions. It has been demonstrated that applying this enzyme to treat orange juice beverages can significantly improve their clarity and taste [185]. In meat processing, protein hydrolytic enzymes also play a crucial role, especially protease hydrolyze muscle and collagen, reducing fiber tightness and enhancing water retention, thereby significantly improving the tenderness and moisture-holding capacity of meat while increasing the solubility of collagen [186]. In addition, using specific proteases, such as recombinant peptidases, to treat meat can effectively hydrolyze collagen, thereby improving the texture of the meat [187]. Specialized enzymes also perform well in the baking industry. For example, using specialized enzyme preparations such as α-amylase, xylanase, and cellulase can hydrolyze polymers like starch, xylan, and cellulose in flour, thereby improving the texture of dough and bread [188].

Compound enzyme preparations show unique advantages when processing more complex food matrices. In beverage processing, the use of compound enzyme preparation can clarify the juice more effectively. For example, during the treatment of pomegranate juice, the combination of protease and pectinase can hydrolyze proteins and degrade pectin, significantly reducing turbidity caused by proteins and phenolic substances, greatly improving the clarity of pomegranate juice while retaining its beneficial compounds [189]. In meat processing, the use of compound enzymes is also advantageous. Combinations of different types of proteases, such as the mixture of plant proteases (bromelain, papain, etc.) and bacterial proteases, can work synergistically to decompose proteins in meat from different sites to achieve the best tenderizing effect [190]. In the baking industry, the use of compound enzyme preparations, such as bread improvers, can enhance the extensibility, moisture retention, and gas bubble stability of dough, thereby significantly improving the rheological properties of the dough and the quality of the bread. For example, *Stachybotrys microspora* produces a complex enzyme with simultaneous starch, xylan, and cellulose decomposing activity in practical applications, which significantly improves dough properties and improves bread quality [191].

The immobilized enzyme preparation, by being fixed onto carriers, achieves high stability and reusability of enzymes, further enhancing the efficiency of food processing. In beverage clarification, the immobilized enzyme is no longer limited to the application of a single enzyme, and the choice of immobilized carrier has been expanded to a variety of new materials, such as Chitosan–Clay Nanocomposite Film [192,193,194]. Benucci et al. fixed bromelsin and pectinase on chitosan beads and applied them in a fluidized-bed reactor to clarify pomegranate juice, which significantly improved the clarifying effect and expanded the application potential of immobilized enzymes in beverage processing [195]. In the bakery industry, immobilized enzymes also show great potential. For example, when glucose oxidase is fixed on zinc oxide nanoparticles, it is used to improve the texture, color, and volume of bread, which not only improves product quality but also provides new ideas for sustainable baking processing [196].

#### 4.2.2. Improve Flavor

Beyond improving quality, enzyme preparations remove undesirable flavors and enhance food taste, with applications in oil refining, meat seasoning, dairy, and beverage production. Lipase, as a specialized enzyme preparation, can gently remove off-flavors and impurities from oils by catalyzing fat hydrolysis, thereby improving the overall quality. During oil refining, factors such as poor raw material quality, oxidation reaction, and microbial decomposition can lead to the presence of free fatty acids, impurities, and volatile compounds in the oil, affecting product quality and flavor [197]. Additionally, tannase breaks down tannins, removing bitterness and clarifying beverages while enhancing color, flavor, and antioxidant properties. It is widely used in beverages like fruit juices, beer, and wine during storage [198]. Tannase can also reduce the formation of “tea cheese” in ready-to-drink teas and help achieve a high content of soluble polyphenol tea in cold water [199].

Compared with the specialized enzyme preparation, compound enzyme preparation can improve the product more efficiently to meet the different production needs. Flavor enzymes, as a standard complex enzyme preparation, can break the hydrophobic cavities that bind proteins to off-flavor compounds through hydrolysis, thereby releasing these compounds and solving the issue of enhanced off-flavors that limit the use of egg white powder in the processing industry [200]. Compound enzyme preparations can also combine hydrolysates with flavor compounds, such as papain, lipase, and flavor protease, which can promote the combination of egg yolk hydrolysates with flavor substances and finally form egg yolk enzymatic hydrolysis products with different flavors [201]. Daqu, as a composite enzyme preparation, is used in the brewing process to provide 90% α-amylase and 99% glucose amylase. For example, in the production of strong-flavored liquor, it acts as a flavoring agent and gradually breaks down macromolecular substances in the raw materials (such as starch, proteins, and esters) into small-molecule flavor compound precursors, thus promoting the formation of baijiu’s unique flavor [202].

In some food processing processes, the use of immobilized enzymes has significant advantages in enhancing food flavor and has better stability, functionality, and recyclability than specialized and compound enzyme preparations. It is worth noting that studies have shown that using chitosan beads as a carrier to immobilize tannase for treating beer wort not only achieves efficient and continuous treatment of beer wort but also significantly improves the stability of the enzyme under broad pH conditions, thus improving the reaction efficiency [203]. Currently, the application of immobilization technology is not limited to beer production. β-glucosidase and α-L-arabinofuranosidase immobilized by comini-CLEAS can effectively break the glucosidase bond of aromatic substances in grapes and release aromatic compounds in wine, such as linol, nerolol, geranyl, etc. These compounds are important factors in determining the typical flavor of Muscat wines [204]. Additionally, immobilized lipoxygenase plays an important role in meat processing, particularly in the oxidation of chicken fat. By using immobilized lipoxygenase, unsaturated fatty acids can be effectively catalyzed to produce lipid oxidation products, which are key to the development of chicken flavor, particularly enhancing the aromatic characteristics of Maillard reaction products [205]. In probiotic yogurt drinks, the use of immobilized glucose oxidase exhibits multiple functions, which not only reduces the concentration of dissolved oxygen, helps maintain the activity and survival rate of probiotics, but also promotes the generation of volatile flavor compounds such as acetaldehyde and diacetyl, thus improving the flavor and texture of yogurt [206].

#### 4.2.3. Promote Fermentation

Enzyme preparations not only enhance flavor and texture but also promote fermentation and boost nutritional value. In rice wine saccharification, enzymes like α-amylase, protease, and glucose amylase convert macromolecules in rice and wheat into glucose, oligosaccharides, and amino acids [207]. Glucose treated with special enzyme preparations can be used as a substrate for alcohol fermentation to promote further fermentation [208]. In addition, legumes contain bioactive compounds such as phenolic acids, flavones, and tannins [209]. However, the anti-nutrient factors, such as protease inhibitors, phytates, and tannins, not only inhibit the activity of digestive enzymes and reduce the absorption of proteins and minerals but also form difficult-to-digest complexes that further hinder the utilization of these nutrients [210]. Through the treatment of special enzyme preparations, the complex proteins in beans can be specifically broken down into small peptides and free amino acids, thereby enhancing their digestibility and nutritional value.

With the deepening research on fermentation processes, the potential of composite enzyme preparations in enhancing fermentation efficiency and nutritional value is gradually emerging. Xu et al. used Alcalase and bromelain to hydrolyze pigeon beans, lentils, and chickpeas and found that after hydrolysis, the degree of hydrolysis increased, the surface hydrophobicity decreased, and the ability of water absorption and oil absorption increased [211]. This provides theoretical support for the production of functional components with beneficial biological activities from legumes by enzymatic hydrolysis. In addition, Pietrzak et al. used the complex enzyme preparation Ceremix 2XL and Ceremix 6X MG to improve the fermentation of wheat-to-rye bread, successfully reducing the fermentation time by 72 h, and the enzyme-treated saccharification solution was more beneficial to the yeast physiology after fermentation. Immobilized enzyme technology also opens up new possibilities for the application of enzyme preparations [212]. Patel et al. injected casein-modified silica into magnetite nanoparticles to fix commercially available alpha-amylase. After 17 cycles of continuous use, the immobilized amylase still retained 52% of residual activity, indicating that it has significant commercial application value [213]. With the continuous development of the food industry and changes in consumer demand, the application of enzyme preparations in food processing will become increasingly important and become an important tool to improve food quality and production efficiency.

### 4.3. Food Preservation

Population growth and food sustainability issues pose significant challenges for developing countries [214]. Meanwhile, an aging population increases the demand for high-nutritional-quality foods, particularly fresh fruits and vegetables. Microbial activity, oxidative reactions, enzymatic action, and inadequate storage conditions contribute to food spoilage, raising the risk of foodborne illnesses and causing economic losses. Many strategies have been implemented to address this issue, including advancements in packaging technology [215], the development of preservation techniques [216], and quality testing [217]. Notably, these technologies heavily rely on enzyme preparations.

Compared to chemical preservation methods, enzyme technologies extend food shelf life without altering sensory properties or nutritional content. These methods are natural and non-toxic, showing promising applications. Currently, the enzymes used to prevent food spoilage mainly originate from microorganisms. Lysozyme ensures food safety by hydrolyzing the β-1,4 glycosidic bonds in bacterial cell walls, leading to microbial death. Its high stability and resistance to acid and heat make it suitable for use in seafood, meat products, and baked goods. Glucose oxidase works by removing oxygen from food, thereby prolonging shelf life by acting on glucose. Additionally, chitinase decomposes bacterial cell walls and improves food texture, while protease extends shelf life through protein hydrolysis and flavor enhancement.

Another approach to food preservation involves the use of innovative preservation materials, natural food preservatives, and smart packaging technologies. This includes chitosan derivatives, nanoemulsions (NEs), and biodegradable food packaging films/coatings (BFPF/BFPC). Recently, Li et al. developed a humidity-responsive antibacterial nanofiber system based on ethylene-vinyl alcohol copolymer (EVOH) [180]. This system incorporates coumarin (CUM) and 2-hydroxypropyl-β-cyclodextrin (HβCD), achieving high entrapment (78%) and loading rates (8.51%), demonstrating significant potential. As a novel smart packaging material, it selectively responds to changes in food or the environment, initially designed for drug delivery systems and sensors. As research progresses, scientists are adding antibacterial or antioxidant agents to these materials. They utilize enzyme-specific reactions to trigger particular chemical reactions, leading to the degradation or release of active components to prevent food spoilage. This approach has been successfully applied in food preservation and crop protection. Additionally, ultrasound proves to be an effective method for animal-derived foods [164].

In summary, the application of enzyme technology in food processing, testing, and preservation, particularly when combined with the advantages of enzyme immobilization technology, not only significantly enhances production efficiency but also fosters sustainable development, reduces reliance on chemical additives, and presents new opportunities for the future of the food industry. Investigating the specific applications of enzyme technology in the food sector and exploring its value holds substantial significance for advancing enzyme technology and ensuring the long-term health of the food processing industry. As enzyme technology evolves, the food industry will become more efficient, safer, and environmentally sustainable, adapting to changing market demands. Ultimately, the advancement of enzyme technology will introduce new opportunities for the food industry and propel comprehensive upgrades and innovations within the sector.

## 5. Conclusions

This paper provides a comprehensive overview of the current research and applications in the field of enzyme engineering, covering topics such as directed evolution, enzyme immobilization technology, the development of novel enzyme sources, and the application of enzyme preparations in food detection, processing, and preservation. This article reviews the history of enzyme research, from the discovery of enzymes to the proposal of directed evolution to the development of high-throughput screening techniques to make enzyme optimization and function enhancement more efficient. It also discusses the development of new enzyme sources obtained in extreme environments, such as “extremozymes” resistant to high temperature, low temperature, strong acid and alkali, and bionic enzymes, which provide more possibilities for the application of enzymes in complex industrial environments. In addition, the development of enzyme immobilization technology and the application of new carrier materials are also introduced, which provide an important means to improve the stability, reusability, and catalytic efficiency of enzymes.

The future development of enzyme engineering will focus on the following directions: First, the introduction of computer-aided design and artificial intelligence technology will greatly improve the efficiency of enzyme-directed evolution and make enzyme design more accurate. At the same time, microbial enzyme sources from extreme environments will become a research hotspot, and their excellent catalytic properties under extreme conditions such as high temperature, low temperature, and strong acid and base provide the possibility to solve the problem of catalysis in complex industrial environments. In addition, future biomimetic enzymes are expected to have higher activity and environmental adaptability than natural enzymes. Further optimization of enzyme immobilization technology will rely on the application of new smart, responsive materials and 3D printing technology, thereby improving the industrial applicability and cost-effectiveness of enzymes. As these advancements continue, enzyme preparations will extend beyond the food industry into diverse applications. Overall, enzyme engineering will develop in a more green, intelligent, and sustainable direction, providing new momentum for the green upgrading of industry and society.

## Figures and Tables

**Table 2:** Advantages and disadvantages of traditional immobilization methods.

Immobilization Method	Whether It Is Reversible or Not	Advantages	Disadvantages	References
Adsorption	Reversible	Low cost, relatively mild conditions, and high enzyme recovery	Poor adsorption specificity, low stability, weak force, and susceptibility to external influences	[68,71]
Entrapment	Irreversible	Maintaining the natural enzyme structure, the carrier material can be modified to create an optimal environment for the enzyme	Mass transfer is limited, easy to leak enzymes, and the carrying capacity is low	[72,73,74]
Covalent bonding	Irreversible	Maintain the relative active conformation and position, and the arrangement of proteins is controllable	High fixation costs, large loss of enzyme activity, structural modification, and/or inactivation	[75]
Cross-linking	Irreversible	High catalytic enzyme activity and high stability	Poor recovery and expensive crosslinkers	[76]

**Table 3:** Commonly used carrier materials for enzyme immobilization and their respective targets.

Material Category	Immobilized Vectors	Immobilize the Object	Reference
Natural polymers	Gelatin	Cellulase	[79]
	Cellulose	Horseradish peroxidase (HRP), glucose oxidase (GOD), catalase (CAT)	[80]
	Alginate-agar gel beads	Pectinase	[81]
	Sodium alginate-β-cyclodextrin	Mannanase	[82]
Synthetic polymers	Chitosan	Bromelain	[83]
	Chitosan-β-cyclodextrin	Keratinase	[84]
Natural and artificially hybridized	Chitosan beads	Mercaptoprotease	[85]
Synthetic polymers	Polyacrylamide gel	Urease	[86]
	Polystyrene microspheres	Lipase	[87]
Inorganic materials	CNTs-Ni	Lipase	[88]
	Fe_3_O_4_-GO	Lipase	[89]
	Alumina	Pectinase	[90]
	SiO_2_	Laccase/lipase	[91,92]

## Data Availability

No new data were created or analyzed in this study. Data sharing is not applicable to this article.
